# Therapeutic potential of CDK11 in cancer

**DOI:** 10.1002/ctm2.1201

**Published:** 2023-02-28

**Authors:** Dalibor Blazek

**Affiliations:** ^1^ Central European Institute of Technology (CEITEC) Masaryk University Brno Czech Republic

**Keywords:** cancer, CDK11, OTS964, pladienolide B, SF3B1, spliceosome, splicing

## Abstract

Human cyclin‐dependent kinases (CDKs) direct the progression of the cell cycle and transcription. They are deregulated in tumours, and despite their involvement in the regulation of basic cellular processes, many CDKs are promising targets for cancer therapy. CDK11 is an essential gene for the growth of many malignancies; however, its primary cellular function has been obscure, and the mode‐of‐action of OTS964, the first CDK11 inhibitor and antiproliferative compound, has been unknown. A recent study has shown that OTS964 prevents spliceosome activation, revealing a key role of CDK11 in the regulation of pre‐mRNA splicing. In light of these findings, we discuss the therapeutic potential of CDK11 in cancer.

## 1 COMMENTARY

Human cyclin‐dependent kinases (CDKs) consist of 21 enzymes that affect various cellular processes. The best understood CDKs play direct roles in the progression of the cell cycle (CDK1, CDK2, CDK4 and CDK6) or transcription (CDK7, CDK8, CDK9, CDK12, CDK13 and CDK19), but the primary cellular functions of the remaining CDKs are largely unknown. Many of the human CDKs are deregulated in tumours, and our understanding of how they individually contribute to the regulation of the cell cycle or transcription in normal and malignant cells has made them attractive anti‐cancer targets. With the CDK4/6 inhibitor palbociclib approved for the treatment of breast cancer and inhibitors of other CDKs that exhibit antiproliferative effects at various stages of preclinical and clinical testing, therapeutic interest in this family of serine‐threonine kinases is growing.^1^, ^2^ However, for many little‐studied CDKs, their therapeutic potential is not fully realized due to a lack of selective inhibitors and missing/incomplete knowledge about their actual substrates and primary cellular functions. The ubiquitously expressed 110 kDa CDK11 is one such underrated CDK. This nuclear enzyme is believed to be involved in transcription, splicing and in the regulation of the cell cycle, apoptosis and autophagy.^3^, ^4^, ^5^ CDK11 is overexpressed in several types of tumours, often predicting a poor clinical outcome. The loss‐of‐function studies indicated a dependency of the growth of several cancers, including osteosarcoma, breast, multiple myeloma and ovarian, on CDK11, and in liposarcoma and ovarian cancer cells it also enhanced the cytotoxic effect of doxorubicin and paclitaxel, respectively.^4^, ^5^ A recent study identified OTS964 as the first CDK11 inhibitor found among drugs in clinical and pre‐clinical development with mischaracterized targets. In agreement with the results of the CDK11 loss‐of‐function studies, the OTS964 decreased the proliferation of several cancer cell lines.^6^ These observations are consistent with a dependency of cancer cells on CDK11 and the potential targeting of the kinase for cancer therapy. Nevertheless, a molecular mode‐of‐action of OTS964 (aka CDK11 substrates) has been an essential unanswered question that hinders our understanding of the anti‐cancer potential of CDK11 inhibition.

**FIGURE 1:**
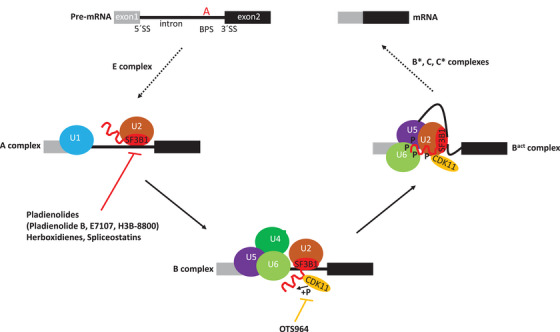
**Spliceosome assembly is blocked at different stages by inhibitors of SF3B1 and CDK11**. The figure represents a simplified scheme of spliceosome assembly on pre‐mRNA consisting of exon 1, exon 2, intron, 5´ and 3´ splice sites (5´SS and 3´SS) and branch point site (BPS) adenosine (A). Five small nuclear ribonucleoprotein particles (snRNPs) (colored U1, U2, U4, U5 and U6 circles), each consisting of several proteins and respective snRNAs, dynamically assemble into various spliceosome complexes. Only spliceosome complexes A, B and B^act^ are depicted, complexes E, B*, C, C* are omitted for simplicity. SF3B1, a core subunit of U2 snRNP, consists of the structured C‐terminal HEAT‐repeat domain (red oval) and the N‐terminal intrinsically disordered threonine‐rich domain (red wavy lane). The HEAT‐repeat domain recognizes branch point site adenosine in the pre‐mRNA in the A complex; this interaction is inhibited by the indicated groups of drugs resulting in blockage of the spliceosome assembly before the formation of the A complex. The threonine‐rich domain is phosphorylated (P) by CDK11 (yellow oval) during the spliceosome transition from the B to B^act^ complex, and the hyperphosphorylated SF3B1 associates with U5 and U6 snRNAs (not shown) within the U5 and U6 snRNPs (violet and green circle, respectively) in the B^act^ complex. The CDK11 inhibitor OTS964 inhibits the phosphorylation of SF3B1 and blocks spliceosome assembly at the B complex stage. CDK11 binds to SF3B1, but it is not known to which spliceosome complex CDK11 is recruited to and released from.

A recent study by Hluchy et al. demonstrated that OTS964 blocks pre‐mRNA splicing in a similar manner to the splicing inhibitor pladienolide B, but via a different mechanism.^7^ Pre‐mRNA splicing is performed by a spliceosome, a huge RNA‐protein complex that is progressively assembled on introns via spliceosome intermediate complexes termed E, A, B, B^act^, B*, C, C*. The intermediates are formed by the stepwise recruitment and release of small nuclear ribonucleoprotein particles (snRNPs) named U1, U2, U4, U5 and U6.^8^ The spliceosome can be blocked at different assembly stages by splicing inhibitors, resulting in aberrant splicing. The OTS964 rapidly (within minutes) inhibits the phosphorylation of a core spliceosome component SF3B1, a subunit of U2 snRNP, and stalls the transition from the precatalytic B to the activated B^act^ spliceosome complex. Mechanistically, CDK11 phosphorylates the N‐terminal threonine residues of SF3B1 that are required for the association of SF3B1 with U5 and U6 snRNAs in the activated form of the spliceosome (B^act^)^7^ (Figure 1). SF3B1 is the most frequently mutated spliceosomal protein in several cancers and is a major drug target. Well‐characterized compounds such as pladienolides, spliceostatins and herboxidienes target the C‐terminal HEAT‐repeat domain of SF3B1, prevent recognition of the branch point site (BPS) at the 3´end of introns by U2 snRNP and block spliceosome assembly before the formation of the A complex (Figure 1). The compounds modulate tumour cell proliferation and several pladienolide B derivatives (e.g., E7107 and H3B‐8800) have been in clinical trials.^9^ These findings, together with the discovered molecular mechanism of CDK11 inhibition,^7^ show that the different protein domains of the SF3B1 can be targeted with different effects on spliceosome assembly, but essentially the same on pre‐mRNA splicing and cellular proliferation and are consistent with a significant anti‐tumour potential of splicing inhibitors (now also including OTS964) and those targeting SF3B1/U2 snRNP specifically.

Considering the key and direct role of CDK11 in the regulation of splicing, what is the anti‐cancer potential of CDK11 inhibition? The therapeutic targeting of splicing takes advantage of vulnerabilities or splicing addictions of cancer cells that arise from their increased dependencies on the core spliceosome machinery. For instance, splicing factor mutation‐bearing cancers are predominantly dependent on the function of the wild‐type spliceosome and are sensitive to the global inhibition of splicing with derivatives of pladienolides, spliceostatins and herboxidienes, resulting in a preferential death of mutant cancer cells. Recurrent, heterozygous and mutually exclusive mutations of splicing factors SF3B1, SRSF2 and U2AF1 frequently occur in blood and solid cancers (for instance in myelodysplastic syndromes, chronic lymphocytic leukaemia, acute myeloid leukaemia, uveal melanoma and breast cancer).^9^ Another example is the targeting of oncogenic transcription factor MYC that regulates gene expression, is involved in cancer cell transformation and growth, and whose overexpression is the most common driver of cancer. MYC by itself is considered undruggable, but MYC‐driven tumours exhibit various dependencies that are essential for cancer survival and can be targeted pharmacologically. Splicing was identified as one such vulnerability. Since the MYC‐driven increase in transcription overloads proper pre‐mRNA splicing, the depletion or pharmacological inhibition of core spliceosome components, including SF3B1, is detrimental for MYC‐dependent cancer cells.^10^ In summary, since splicing factor‐mutated and MYC‐driven tumours are more sensitive to splicing modulation, CDK11 inhibition could represent a novel synthetically lethal interaction for such splicing‐dependent cancers. As with drugs targeting SF3B1 or the U2 snRNP complex, OTS964 also reciprocally affects RNA polymerase II‐mediated transcription.^7^, ^11^ Therefore, it will be relevant to determine whether OTS964 can also target malignancies dependent on other oncogenic transcription factors and/or synergize with inhibitors of transcriptional CDKs. Another avenue for exploration the therapeutic potential of CDK11 inhibition stems from the discovery of splicing modulation enhancing anti‐tumour immunity. Mice treated with splicing inhibitors induced neoantigens that augmented the response to immune check point blockage with anti‐Programmed cell death protein 1 (PD1) in a manner dependent on T cells, and slowed tumour growth.^12^

Given the myriad described cellular functions affected directly (or indirectly) by CDK11,^3^, ^4^, ^5^, ^7^ there are likely many other ways of targeting CDK11 in different cancer contexts. To obtain a clear rationale for other therapeutic interventions, we need a better understanding of the precise molecular mechanisms of CDK11 actions. With a selective CDK11 inhibitor in hand and the established role of CDK11 in splicing, we can now study the other CDK11‐dependent cellular processes. This will facilitate the rational design of other therapeutic strategies.

## CONFLICT OF INTEREST STATEMENT

The author declares no conflict of interests.

## References

[1] Parua PK , Fisher RP . Dissecting the Pol II transcription cycle and derailing cancer with CDK inhibitors. Nat Chem Biol. 2020;16:716‐724. 32572259 10.1038/s41589-020-0563-4 PMC7914107

[2] Chou J , Quigley DA , Robinson TM , Feng FY , Ashworth A . Transcription‐associated cyclin‐dependent kinases as targets and biomarkers for cancer therapy. Cancer Discov. 2020;10:351‐370. 32071145 10.1158/2159-8290.CD-19-0528

[3] Gajduskova P , Mozos IRL , Rájecký M , Hluchý M , Ule J , Blazek D . CDK11 is required for transcription of replication‐dependent histone genes. Nat Struct Mol Biol. 2020;27:500‐510. 32367068 10.1038/s41594-020-0406-8 PMC7116321

[4] Loyer P , Trembley JH . Roles of CDK/Cyclin complexes in transcription and pre‐mRNA splicing: cyclins L and CDK11 at the cross‐roads of cell cycle and regulation of gene expression. Semin Cell Dev Biol. 2020;107:36‐45. 32446654 10.1016/j.semcdb.2020.04.016

[5] Zhou Y , Shen JK , Hornicek FJ , Kan Q , Duan Z . The emerging roles and therapeutic potential of cyclin‐dependent kinase 11 (CDK11) in human cancer. Oncotarget. 2016;7:40846‐40859. 27049727 10.18632/oncotarget.8519 PMC5130049

[6] Lin A , Giuliano CJ , Palladino A , et al. Off‐target toxicity is a common mechanism of action of cancer drugs undergoing clinical trials. Sci Transl Med. 2019;11:eaaw8412. 31511426 10.1126/scitranslmed.aaw8412 PMC7717492

[7] Hluchy M , Gajdušková P , Mozoset IRL , et al. CDK11 regulates pre‐mRNA splicing by phosphorylation of SF3B1. Nature. 2022;609:829‐834. 36104565 10.1038/s41586-022-05204-z

[8] Wilkinson ME , Charenton C , Nagai K . RNA splicing by the spliceosome. Annu Rev Biochem. 2020;89:359‐388. 31794245 10.1146/annurev-biochem-091719-064225

[9] Stanley RF , Abdel‐Wahab O . Dysregulation and therapeutic targeting of RNA splicing in cancer. Nat Cancer. 2022;3:536‐546. 35624337 10.1038/s43018-022-00384-z PMC9551392

[10] Hsu TY , Simon LM , Neill NJ , et al. The spliceosome is a therapeutic vulnerability in MYC‐driven cancer. Nature. 2015;525:384‐388. 26331541 10.1038/nature14985 PMC4831063

[11] Caizzi L , Monteiro‐Martins S , Schwalb B , et al. Efficient RNA polymerase II pause release requires U2 snRNP function. Molecular cell. 2021;81(9):1920‐1934.e9. 33689748 10.1016/j.molcel.2021.02.016

[12] Lu SX , Monteiro‐Martins S , Schwalb B , et al. Pharmacologic modulation of RNA splicing enhances anti‐tumor immunity. Cell. 2021;184:4032‐4047.e4031 . 34171309 10.1016/j.cell.2021.05.038 PMC8684350

